# Public perceptions of responsibility for recommended food policies in seven countries

**DOI:** 10.1093/eurpub/ckad020

**Published:** 2023-02-10

**Authors:** Ana-Catarina Pinho-Gomes, Leon Booth, Simone Pettigrew

**Affiliations:** The George Institute for Global Health, School of Public Health, Imperial College London, London, UK; Institute of Health Informatics, University College London, London, UK; The George Institute for Global Health, University of New South Wales, Sydney, New South Wales, Australia; The George Institute for Global Health, University of New South Wales, Sydney, New South Wales, Australia

## Abstract

**Background:**

Food policy is important to promote healthy and sustainable diets. However, who is responsible for developing and implementing food policy remains contentious. Therefore, this study aimed to investigate how the public attributes responsibility for food policy to governments, individuals and the private sector.

**Methods:**

A total of 7559 respondents from seven countries [Australia (*n* = 1033), Canada (*n* = 1079), China (*n* = 1099), India (*n* = 1086), New Zealand (*n* = 1090), the UK (*n* = 1079) and the USA (*n* = 1093)] completed an online survey assessing perceived responsibility for 11 recommended food policies.

**Results:**

Overall, preferred responsibility for the assessed food policies was primarily attributed to governments (62%), followed by the private sector (49%) and individuals (31%). Respondents from New Zealand expressed the highest support for government responsibility (70%) and those from the USA the lowest (50%). Respondents from the USA and India were most likely to nominate individuals as responsible (both 37%), while those from China were least likely (23%). The private sector had the highest attributed responsibility in New Zealand (55%) and the lowest in China and the USA (both 47%). Support for government responsibility declined with age and was higher among those on higher incomes, with a university degree, and who perceived themselves to consume a healthy diet or be in poor health.

**Conclusions:**

Across seven diverse countries, results indicate the public considers government should take primary responsibility for the assessed food policies, with modest contribution from the private sector and minority support for individual responsibility.

## Introduction

Suboptimal diet is one of the leading risk factors for the global burden of disease and a major contributor to morbidity and mortality due to non-communicable diseases.^1^ Food-based dietary guidelines have been developed by national and international agencies based on observational and trial data linking certain foods and nutrients to health outcomes.^2^ However, dietary guidelines alone are unable to lead to substantial behaviour change in the absence of an environment that promotes healthy eating.^3^ There is compelling evidence that achieving effective individual behaviour change related to diet requires structural approaches addressing the interlinked components of the obesogenic environment.^4^ In response, some governments are adopting evidence-based food policies that address these structural elements, such as by taxing sugar-sweetened beverages, placing restrictions on unhealthy food advertisements that target children and regulating the salt content of foods.^5^^,^^6^

Despite the benefits of such policies for population health, many governments remain reluctant to implement food policies due to concerns about a lack of public support and industry resistance.^7^ This reflects the ongoing tensions between individual sovereignty, market freedom and common good, which are at the core of public health policy.^8^ Governments attempting to be proactive in this space can face accusations of imposing a ‘nanny state’ where interventionist approaches violate individual freedoms.^9^^,^^10^ This argument seems flawed in the context of public health, where governments are ethically obliged to intervene to protect the liberty of individuals who are being misinformed and manipulated by industry, thus counteracting ‘nefarious nannying’ by commercial actors and enhancing individual liberty.^11^

Although lack of public support for government intervention to regulate, shape and control the food environment is often cited to justify inertia in enacting much-needed food policy,^12^ it is uncertain to what extent this reflects reality. Instead, this argument might be hiding vested interests and lobbying from the industry and deflecting responsibility to individuals.^13^ To provide insights into this situation, this study aimed to investigate whether the public would prefer to assign responsibility for various food policies to governments, individuals, and/or the private sector across seven countries. The examined policies covered food availability and affordability, food advertising, provision of information about healthy eating, improving food composition and making sure food systems are environmentally friendly.

## Methods

This study was part of an international online survey project assessing consumer responses to a range of food and beverage policies.^14^^,^^15^ In 2019, an ISO-accredited web panel provider (Pureprofile) was commissioned to collect data from about 1000 adults from each of seven countries (Australia, Canada, China, India, New Zealand, the UK, and the USA; total = 7559). This sample size was selected for feasibility considerations and irrespective of the population size of each country due to the discrepancy in population sizes between countries. The probability proportional to size method for the selection of the subjects was not applied as this would have generated very different samples sizes, which would have rendered comparisons between countries difficult. Pureprofile used various recruitment strategies to populate its global panel, including mass media advertising, online advertising, mall intercept interviews and word-of-mouth referrals. Quotas were applied to generate samples characterized by approximately even sex and age (18–34, 35–54, 55+ years) distribution, and around two-thirds of the sample representing low- and middle-income households. No quotas were applied for area of residence (i.e. urban vs. rural residents). Individuals completing the survey received small financial reimbursements according to the rates applicable in their respective countries. The study received approval from a University Human Research Ethics Committee.

In addition to a range of demographic questions, such as those relating to gender, age, household income and education, respondents were asked to report their perceptions of their overall health and the healthiness of their diets. Respondents were also asked whether they thought various types of entities [government, the private sector (i.e. companies and commercial organisations), and individuals] should be responsible for 11 policies relating to the affordability and accessibility of healthy food, regulation of unhealthy food advertising and marketing, food composition and reformulation, and food sustainability (detailed description in Supplementary table S1). These policies were selected based on interventions targeting unhealthy diets recommended by the World Health Organization (WHO) to reduce modifiable risk factors for non-communicable diseases and address underlying social determinants through the creation of health-promoting environments.^16^ Respondents could select multiple actors for each action or select the option ‘No-one because it is not important’. The surveys administered in China and India were translated into Mandarin and Hindi, respectively, with an English version also available.

### Data analysis

Descriptive analyses were performed to assess the level of responsibility attributed to governments, individuals and the private sector overall and stratified by country for each of the 11 policies separately and aggregated together. Differences between countries for attributed responsibility across the policies were assessed using Chi-squared tests for differences between proportions. Pairwise comparisons were performed, and the Bonferroni–Holm method was used to calculate *P*-values adjusted for multiple comparisons.

Shared responsibility was calculated as the average number of agents identified as responsible for each policy by each respondent. This allowed comparing to what extent each policy was considered to be within the remit of multiple stakeholders between countries. One-way analysis of variance and Tukey *post hoc* tests were used to test for differences in shared responsibility between countries. Multivariate linear regression models were developed to estimate the association between overall support for government responsibility for food policies and individual characteristics. Overall support for government responsibility was calculated as the average across the 11 policies. Mixed effects models were used to account for clustering of participants within countries. All models were adjusted for the following independent variables: sex (female vs. male), age (continuous), household income level (lower, middle, and upper thirds), education (university education vs. lower education), self-reported health (poor/fair vs. good/very good health) and perceived healthiness of diet (unhealthy vs. healthy). All *P*-values were calculated from two-tailed tests and considered statistically significant if under 0.05. All analyses were performed using R version 4.0.3.

## Results

Overall, this study included 7559 participants, of whom 3802 (50.3%) were women. The average age was 44.7 years (SD 16.6). There were 1033 participants from Australia, 1079 from Canada, 1099 from China, 1086 from India, 1090 from New Zealand, 1079 from the UK and 1093 from the USA. The demographic characteristics of the participants stratified by country are summarised in Supplementary table S2.

### Overall responsibility for food policy

Across the aggregated policies, responsibility was primarily attributed to governments (62%), followed by the private sector (49%) and individuals (31%). Although this trend was observed across all countries, there were significant differences in the distribution of responsibility between countries (table 1). Respondents from New Zealand expressed the highest support for government responsibility (70%), followed by the UK (66%) and Canada (66%). Those from the USA exhibited the lowest support for government responsibility (50%). By contrast, the countries where respondents were more likely to nominate individuals as responsible were the USA and India (37% for both), whilst respondents from China were the least likely to identify individuals as responsible (23%). Support for the private sector being responsible was highest in New Zealand (55%) and Australia (51%), and lowest in China and the USA (47% for both).

**Table 1:** Overall attribution of responsibility for food policies to governments, individuals and private sector across seven countries

Country	Governments	Individuals	Private sector	Not important
Australia (*n* = 1033)	64.4^a^	30.6^a^	50.5^a^	7.0^a^
Canada (*n* = 1079)	66.3^b^	30.5^a^	48.2^bcd^	7.1^a^
China (*n* = 1099)	53.2^c^	22.7^b^	46.6^b^	1.5^b^
India (*n* = 1086)	63.7^a^	36.7^c^	48.7^ac^	2.3^c^
New Zealand (*n* = 1090)	69.7^d^	31.9^a^	55.4^e^	5.5^d^
United Kingdom (*n* = 1079)	66.4^b^	25.1^d^	48.5^cd^	5.5^d^
United States (*n* = 1093)	50.2^e^	36.8^c^	46.9^bd^	9.5^e^
Total (*n* = 7559)	62.0	30.6	49.2	5.5

Numbers represent the average percentage of respondents who attributed responsibility to each agent across all food policies included in the survey.

Different letters represent statistically significant differences based on an adjusted *P* values below 0.05 for differences in percentages of ‘yes’ answers between countries; proportions sharing a superscript number are not significantly different from each other, whereas proportions with different superscript numbers are significantly different from each other.

Overall, the percentage of respondents considering policies as not important was 5.5% (table 1). Respondents were more likely to identify a policy as not important in the USA (9.5%), followed by Canada and Australia (7% for both). The percentage of respondents considering policies as not important was low in China (1.5%) and India (2.3%).

On average, participants attributed responsibility to 1.42 actors among the three categories of governments, individuals and the private sector. Shared responsibility was the highest among respondents from New Zealand, with an average of 1.57 agents selected per policy, and the lowest in China, with an average of 1.23 (table 2). Shared responsibility also varied across policies, ranging from 1.14 for ‘Deciding how many fast-food outlets are allowed in specific areas’ to 1.71 for ‘Making sure healthy foods and beverages are available for everyone’.

**Table 2:** Shared responsibility stratified by country and question

Country	Making sure healthy foods and beverages are available for everyone	Limiting the amount of junk-food advertising	Making healthy foods available in workplaces	Making sure healthy foods are affordable	Broadcasting public education campaigns about healthy eating	Deciding what nutrition information should be on food products	Deciding how many fast-food outlets are allowed in specific areas	Ensuring foods are reformulated to make them healthier	Setting targets for the amount of fat, sugar and salt in packaged foods	Making sure children are not exposed to marketing for unhealthy foods	Making sure food production and distribution processes are environmentally friendly	Overall
Australia	1.82	1.41	1.52	1.53	1.37	1.45	1.12	1.36	1.39	1.47	1.57	1.45^bc^
Canada	1.79	1.32	1.50	1.56	1.39	1.45	1.08	1.42	1.42	1.42	1.61	1.45^bc^
China	1.38	1.19	1.23	1.22	1.18	1.22	1.10	1.22	1.19	1.24	1.30	1.23^e^
India	1.68	1.46	1.51	1.49	1.48	1.50	1.33	1.45	1.41	1.47	1.60	1.49^ab^
New Zealand	2.00	1.54	1.61	1.63	1.47	1.58	1.20	1.43	1.45	1.64	1.73	1.57^a^
UK	1.69	1.39	1.39	1.48	1.30	1.42	1.17	1.31	1.33	1.42	1.49	1.40^cd^
US	1.60	1.18	1.38	1.47	1.33	1.40	0.99	1.29	1.31	1.28	1.49	1.34^d^
Total	1.71	1.35	1.45	1.48	1.36	1.43	1.14	1.35	1.36	1.42	1.54	1.42

Numbers represent the average number of stakeholders each respondent considered responsible for each policy (out of three possible stakeholders). The larger the number, the more responsibility was considered to be shared between stakeholders.

Different letters represent statistically significant differences between countries based on an adjusted *P* values under 0.05 for pairwise comparisons between countries; proportions sharing a superscript number are not significantly different from each other, whereas proportions with different superscript numbers are significantly different from each other.

### Responsibility for different food policies

**Figure 1:**
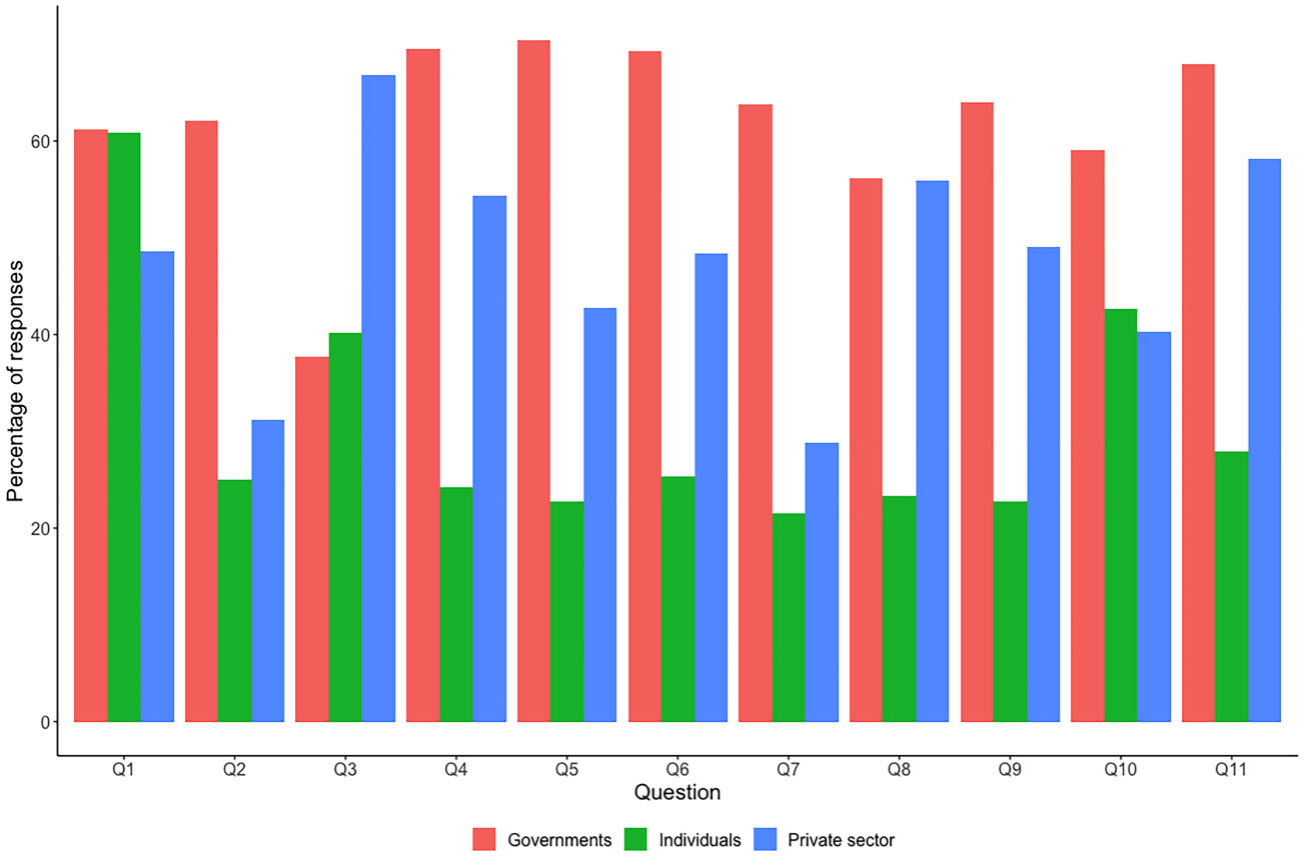
Overall attributed responsibility to governments, individuals and the private sector for 11 food policies. q1: Making sure healthy foods and beverages are available for everyone; q2: Limiting the amount of junk-food advertising; q3: Making healthy foods available in workplaces; q4: Making sure healthy foods are affordable; q5: Broadcasting public education campaigns about healthy eating; q6: Deciding what nutrition information should be on food products; q7: Deciding how many fast-food outlets are allowed in specific areas; q8: Ensuring foods are reformulated to make them healthier; q9: Setting targets for the amount of fat, sugar and salt in packaged foods; q10: Making sure children are not exposed to marketing for unhealthy foods; q11: Making sure food production and distribution processes are environmentally friendly

The aggregate responsibility attributed to each actor varied substantially across policies (figure 1). Across the seven countries, the highest level of support for government intervention was for ‘Broadcasting public education campaigns about healthy eating’ (71%), ‘Making sure healthy foods are affordable’ (70%), and ‘Deciding what nutrition information should be on food products’ (69%) (Supplementary table S3). The highest level of support for individual responsibility was found for ‘Making sure healthy foods and beverages are available for everyone’ (61%) followed by ‘Making sure children are not exposed to marketing for unhealthy foods’ (43%) (Supplementary table S4). For the private sector, greatest support was found for ‘Making healthy foods available in workplaces’ (67%) followed by ‘Making sure food production and distribution processes are environmentally friendly’ (58%) (Supplementary table S5).

There was wide variation between countries in the allocation of responsibility to government, individuals and the private sector across the assessed policies. For instance, in the USA, government responsibility for advertising was only 40%, which was much lower than 71% in the UK and New Zealand. In New Zealand, 85% of respondents considered government to be an appropriate entity for making sure healthy foods are affordable, in comparison to only 53% in China. Similarly, individuals were considered responsible for ‘Making sure healthy foods and beverages are available for everyone’ by 77% of respondents in New Zealand compared to only 43% in China. In India, 38% of respondents considered individuals responsible for ‘Making sure food production and distribution processes are environmentally friendly’ in comparison to 20% in China and 21% in the UK. ‘Deciding how many fast-food outlets are allowed in specific areas’ was reported to be a private sector responsibility by 37% of respondents in India and 36% in China in comparison to about 25% in Australia, Canada and the UK. The private sector was considered more responsible for ‘Making sure food production and distribution processes are environmentally friendly’ in New Zealand (71%) than in other countries, particularly China (52%), India (54%) and the USA (54%).

Overall, the percentage of respondents considering policies as not important was under 10% for all policies (Supplementary table S6), apart from ‘Deciding how many fast-food outlets are allowed in specific areas’ (13% across all countries). The percentage of respondents considering this policy as not important was highest in the USA (27%) and Canada (20%).

### Individual characteristics associated with greater support for government responsibility

**Figure 2:**
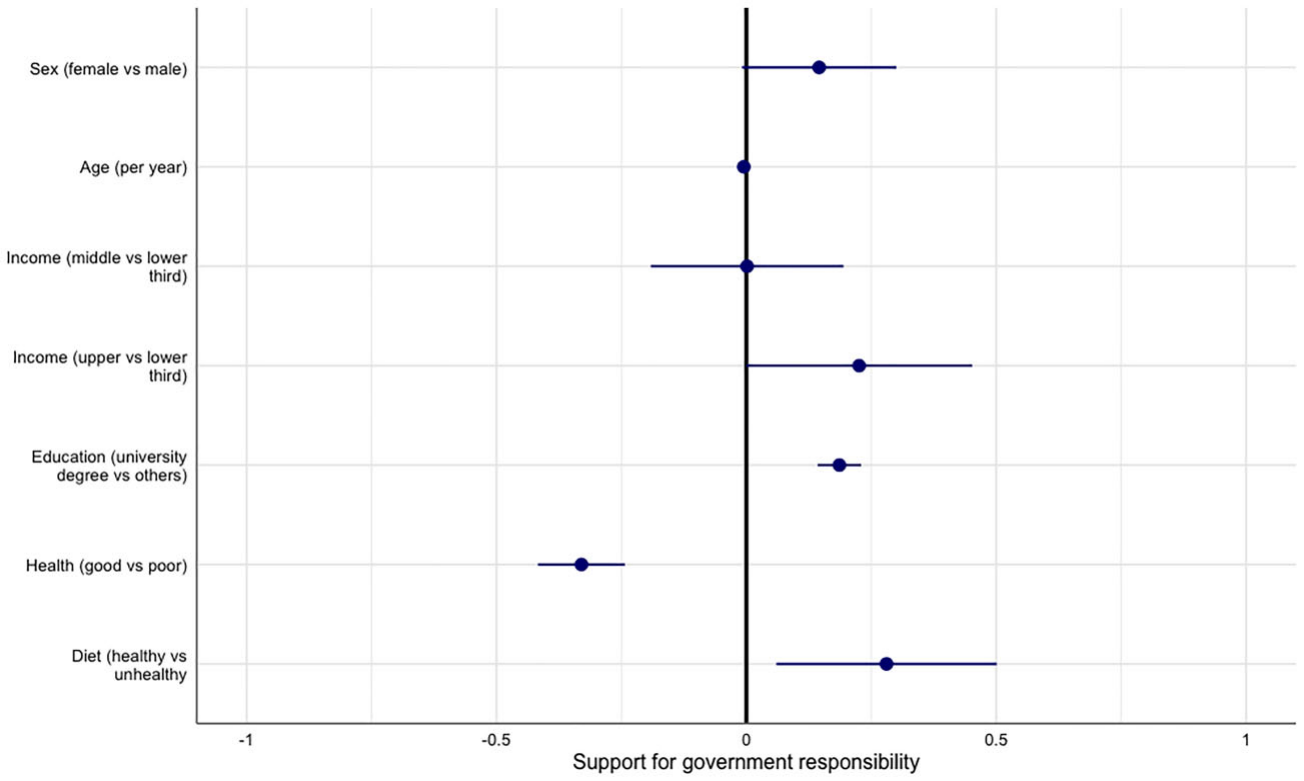
Factors associated with greater support for government responsibility for food policy. Forest plot shows results of multivariate linear regression (based on mixed effects model with clustering by country). Dots represent estimates and lines represent 95% confidence intervals. Factors that are significantly associated with increased (positive, right) or reduced (negative, left) support for government responsibility are those for which the 95% confidence interval does not include the value zero

Respondent characteristics significantly associated with attributing greater responsibility to governments for food policy were being in the upper third of the income distribution for their respective countries (23% higher than lower income third), having a university degree (19% higher than not having a university degree) and perceiving they consume a healthy diet (28% higher than perceiving an unhealthy diet) (figure 2 and Supplementary table S7). The characteristics significantly associated with considering governments less responsible for food policy were age (0.5% reduction per year) and self-reporting as being in good health vs. poor/fair health (33% reduction).

## Discussion

This study found that across seven different countries, the general public considered food policies as important and identified government as the appropriate actor to take responsibility for a range of evidence-based food policies covering availability and affordability of food, advertising of unhealthy foods (including marketing to children), provision of information about healthy eating, regulation of food composition and ensuring the environmental sustainability of the food system. The strong support for government action in these areas suggests policy makers may have legitimacy to introduce those policies that have not been implemented to date. However, there were marked differences between countries. The USA was the country where respondents were more likely to identify food policies as not important. The greatest support for government intervention was found in New Zealand and the lowest in the USA. Whilst government was nominated as the primary responsible agent for most food policies, government responsibility was higher for policies related to education campaigns, regulation of marketing and food labelling, accessibility and affordability of healthy food and environmental sustainability of food. The private sector had substantial responsibility attributed for availability of healthy food in the workplace, food reformulation, environmental sustainability of food and affordability of healthy food. Individuals were considered, in general, to have modest responsibility, other than for ensuring everyone had access to healthy food. Support for government responsibility was higher among those who had higher incomes, completed a university degree, and perceived themselves to consume a healthy diet. On the contrary, attribution of responsibility to government declined with age and was lower in those reporting good health in comparison to those in poor health.

The highest support for government intervention was observed for broadcasting information campaigns, which is in keeping with previous evidence suggesting that people prefer policies that are less intrusive into personal life and less restrictive of individual freedoms.^17^ Although education on healthy eating is important, information alone is unable to tackle the problem of unhealthy diets due to intrinsic and extrinsic barriers.^18^ Evidence has shown that changing the environment (i.e. focusing on opportunity) is paramount to improving diets.^19^ This includes policies related to both affordability and accessibility of healthy food, as cost remains a key determinant of food choices.^20^ Support for governments being responsible for ensuring healthy food is accessible and affordable to all was high in all countries except China and the USA, yet this responsibility was commonly shared with the private sector. This may indicate the public wants governments and companies to work in tandem to ensure healthy foods are available and affordable for everyone, thus reducing inequalities in diet-related diseases.^21^ First, governments are responsible for developing policy that ensure healthy food is fairly priced or subsidized.^22^ Then, food producers, manufacturers, distributers and sellers are responsible for enacting those policies.^23^

There was broad acknowledgment that governments are responsible for advertising regulation and control, particularly in relation to children and young people, who are especially vulnerable to deleterious influences.^24^ The USA, well known for having a more libertarian and market-driven society, was the country with the lowest support for marketing regulation by government (40% compared to over 70% for New Zealand and the UK). By conveying conflicting messages to the public, the ubiquitous advertising of unhealthy foods can jeopardize the success of public health information campaigns.^25^ Therefore, marketing restrictions, such as limiting child-targeted advertising of foods and beverages that do not comply with basic nutrition guidelines, are recommended by the WHO.^16^ Notwithstanding the intense debate surrounding the implementation of such policies and fierce resistance from industry and libertarian politicians,^26^ the results of the present study indicate that half of those in the USA and the majority of the general public in the other included countries expect government to take responsibility for protecting people, and especially children, from unhealthy food marketing.

Although governments were nominated as the primary responsible actors for food reformulation, the private sector was also identified as a key player in this area. This may reflect an understanding that governments can nudge or, less often, mandate companies to reformulate their products, particularly processed foods, through strategies such as applying taxes or setting limits on food composition.^27^ However, the research and innovation behind food reformulation depend on the industry. Food reformulation can reduce intake of potentially harmful nutrients, such as sugar, salt and trans-fats, without forcing people to change consumption of the foods they enjoy or compromising sales, and hence profits, of companies, as demonstrated by the reformulation outcomes of the sugar drinks industry levy introduced in the UK in 2018.^28^ Tough restrictions on food composition, such as banning artificial trans-fats from processed foods, may also prompt and accelerate replacement of those ingredients by healthier options.^29^ Therefore, food reformulation strategies may be particularly suited to countries, such as China and the USA, where there was less support for governments to regulate food labelling and composition, perhaps due to concerns about the potential impact on the food industry, international trade and the economy.^30^

Environmental sustainability has been brought to the fore of the world’s agenda, with rising concerns about how current food systems and diets are incompatible with meeting carbon targets and global warming goals.^31^ Our results suggest that governments, followed by the private sector, are likely to be perceived as responsible for ensuring food systems are sustainable. This perhaps reflects recognition that individual-level actions have a relatively minor impact unless structural changes are introduced at national and international levels to reduce the carbon footprint of food production, processing, and distribution. The lower support for government responsibility for food sustainability in China and the USA in comparison to New Zealand, the UK and Canada hints at differing societal values and environmental priorities.

Finally, it is important to emphasize that no single intervention can tackle the complexity of the global food system, and different approaches can be complementary and synergistic.^32^ Therefore, successfully changing our diets for the sake of human and planetary health requires comprehensive strategies, intersectoral action and integration between stakeholders at local, national, and international levels.^33^ This should involve upstream measures focused on food production, transport, processing and marketing as well as mid and downstream measures at the consumer level, including schools, workplaces, healthcare settings and homes.^34^ Such a ‘food and health in all policies’ approach relies on strong government leadership,^27^ and this study indicates the public recognizes and sanctions the critical role governments play in changing food systems. Understanding our unhealthy and unsustainable food system as a societal rather than an individual problem may help sharing responsibility between governments, individuals and the private sector.

This study has important strengths. First, it included a large sample of participants from seven countries that are culturally, economically, and politically distinct. Second, it covered a wide range of policies, which were based on WHO recommendations for cost-effective interventions to reduce the impact of unhealthy diets. However, there are also some limitations to consider when interpreting the results. First, the samples were recruited via a web panel provider and may thus have different psychographic characteristics compared to other samples. Although quotas were used for some variables, findings may not be generalizable to the populations of those countries. Second, the quantitative study design facilitated assessment of which agents and combinations of agents were considered responsible for implementing several food policies but did not allow exploration of how respondents perceived responsibility to be shared by different actors or understanding the reasons underlying their choices. Future research could explore if and how members of the public conceive optimal cooperation and coordination between actors.

## Conclusions

This study suggests there may be broad public support in the seven diverse countries for governments to take decisive action on matters related to the availability and affordability of healthy food, food composition and marketing and environmental sustainability. The private sector was also identified as a key stakeholder, particularly for food composition and environmental sustainability, whilst individuals were considered to have the lowest responsibility. These results indicate that communities may be supportive of governments introducing evidence-based policies and, where appropriate, collaborating with the private sector to implement them. Therefore, the public seems to consider government should take primary responsibility for food policy and regulation, with modest contribution from the private sector and minority support for individual responsibility.

## Supplementary Material

ckad020_Supplementary_Data

## Data Availability

Data are available upon request from the corresponding author.
