# Supplementary material for: Public perceptions of responsibility for recommended food policies in seven countries
Source: Eur J Public Health. 2023 Feb 10;33(2):299–304. doi: 10.1093/eurpub/ckad020 (PMC10066496; doi:10.1093/eurpub/ckad020)
Supplement: ckad020_Supplementary_Data [file ckad020_supplementary_data.docx]

**Is there appetite for government intervention in the food system?**

**Supplementary data**

**Table S1: Food policies and stakeholders**

| Policies | Stakeholders |
| --- | --- |
| Making sure healthy foods and beverages are available for everyone | Individuals |
| Limiting the amount of junk-food advertising | Governments |
| Making healthy foods available in workplaces | Private sector (i.e., companies/commercial organisations) |
| Making sure healthy foods are affordable |  |
| Broadcasting public education campaigns about healthy eating |  |
| Deciding what nutrition information should be on food products |  |
| Deciding how many fast-food outlets are allowed in specific areas |  |
| Ensuring foods are reformulated to make them healthier |  |
| Setting targets for the amount of fat, sugar, and salt in packaged foods |  |
| Making sure children are not exposed to marketing for unhealthy foods |  |
| Making sure food production and distribution processes are environmentally friendly |  |

**Table S2: Demographic characteristics of the participants included from each country**

| Demographic characteristics | Australia  (n=1033) | Canada  (n=1079) | China  (n=1099) | India  (n=1086) | New Zealand  (n=1090) | United Kingdom  (n=1079) | United States  (n=1093) | Total sample  (n=7559) |
| --- | --- | --- | --- | --- | --- | --- | --- | --- |
|  | **%** | **%** | **%** | **%** | **%** | **%** | **%** | **%** |
| Sex |  |  |  |  |  |  |  |  |
| Female | 51 | 51 | 49 | 48 | 52 | 51 | 51 | 50 |
| Male | 49 | 49 | 51 | 52 | 48 | 49 | 49 | 50 |
| Age (years) |  |  |  |  |  |  |  |  |
| 18-34 | 31 | 29 | 32 | 48 | 30 | 29 | 30 | 33 |
| 35-54 | 34 | 34 | 42 | 34 | 39 | 35 | 34 | 36 |
| 55+ | 35 | 37 | 26 | 18 | 31 | 36 | 36 | 31 |
| Household income |  |  |  |  |  |  |  |  |
| Low | 34 | 33 | 13 | 29 | 28 | 33 | 42 | 29 |
| Mid | 36 | 49 | 63 | 58 | 42 | 28 | 41 | 46 |
| High | 30 | 18 | 24 | 13 | 30 | 40 | 16 | 25 |
| Education |  |  |  |  |  |  |  |  |
| Up to technical degree | 61 | 61 | 45 | 17 | 55 | 58 | 58 | 51 |
| University degree | 38 | 39 | 54 | 82 | 43 | 40 | 41 | 48 |
| Self-rated health |  |  |  |  |  |  |  |  |
| Excellent | 5 | 4 | 0 | 1 | 4 | 7 | 3 | 3 |
| Very good | 23 | 21 | 16 | 12 | 19 | 23 | 19 | 19 |
| Good | 43 | 47 | 39 | 37 | 47 | 39 | 41 | 42 |
| Fair | 43 | 47 | 39 | 37 | 47 | 39 | 41 | 42 |
| Poor | 25 | 23 | 38 | 29 | 26 | 24 | 28 | 27 |
| Diet |  |  |  |  |  |  |  |  |
| Very healthy | 1 | 2 | 0 | 37 | 1 | 2 | 3 | 7 |
| Mostly healthy | 14 | 19 | 8 | 56 | 14 | 18 | 23 | 22 |
| Mostly unhealthy | 69 | 70 | 72 | 6 | 77 | 68 | 60 | 60 |
| Very unhealthy | 15 | 9 | 19 | 1 | 7 | 12 | 14 | 11 |

**Table S3: Percentage of individuals attributing responsibility to governments stratified by country and policy**

| Country | Making sure healthy foods and beverages are available for everyone | Limiting the amount of junk-food advertising | Making healthy foods available in workplaces | Making sure healthy foods are affordable | Broadcasting public education campaigns about healthy eating | Deciding what nutrition information should be on food products | Deciding how many fast-food outlets are allowed in specific areas | Ensuring foods are reformulated to make them healthier | Setting targets for the amount of fat, sugar, and salt in packaged foods | Making sure children are not exposed to marketing for unhealthy foods | Making sure food production and distribution processes are environmentally friendly |
| --- | --- | --- | --- | --- | --- | --- | --- | --- | --- | --- | --- |
| Australia | 61.1 | 66.5 | 35.0 | 71.0 | 75.4 | 75.5 | 67.9 | 57.4 | 68.7 | 60.8 | 69.0 |
| Canada | 67.9 | 60.3 | 37.2 | 77.7 | 76.6 | 78.9 | 62.3 | 62.8 | 68.8 | 61.4 | 75.3 |
| China | 53.4 | 62.1 | 41.9 | 53.4 | 58.8 | 45.6 | 60.0 | 46.6 | 49.0 | 57.1 | 57.8 |
| India | 67.5 | 63.6 | 51.0 | 67.3 | 67.9 | 63.4 | 67.5 | 61.7 | 63.2 | 59.2 | 68.5 |
| NZ | 66.6 | 71.0 | 30.6 | 84.5 | 83.2 | 83.2 | 73.2 | 58.3 | 74.9 | 67.8 | 73.2 |
| UK | 61.3 | 71.3 | 39.1 | 72.5 | 74.8 | 73.9 | 72.8 | 57.7 | 71.2 | 65.2 | 70.8 |
| US | 51.1 | 40.4 | 29.4 | 60.9 | 57.1 | 65.3 | 43.5 | 48.4 | 53.0 | 41.9 | 60.9 |
| Total | 61.3 | 62.2 | 37.7 | 69.6 | 70.5 | 69.4 | 63.9 | 56.1 | 64.1 | 59.1 | 67.9 |

Numbers represent the % of respondents who attributed responsibility to governments

**Table S4: Percentage of individuals attributing responsibility to individuals stratified by country and policy**

| Country | Making sure healthy foods and beverages are available for everyone | Limiting the amount of junk-food advertising | Making healthy foods available in workplaces | Making sure healthy foods are affordable | Broadcasting public education campaigns about healthy eating | Deciding what nutrition information should be on food products | Deciding how many fast-food outlets are allowed in specific areas | Ensuring foods are reformulated to make them healthier | Setting targets for the amount of fat, sugar, and salt in packaged foods | Making sure children are not exposed to marketing for unhealthy foods | Making sure food production and distribution processes are environmentally friendly |
| --- | --- | --- | --- | --- | --- | --- | --- | --- | --- | --- | --- |
| Australia | 68.8 | 24.1 | 46.8 | 23.2 | 18.1 | 22.2 | 19.3 | 21.5 | 20.0 | 45.9 | 26.3 |
| Canada | 61.4 | 24.3 | 42.6 | 22.7 | 21.0 | 24.9 | 20.5 | 21.9 | 23.4 | 44.9 | 27.7 |
| China | 43.4 | 14.7 | 23.4 | 18.9 | 22.5 | 25.5 | 14.0 | 21.1 | 19.2 | 26.9 | 20.3 |
| India | 54.6 | 33.5 | 40.0 | 33.7 | 33.6 | 34.3 | 28.5 | 32.9 | 29.3 | 45.1 | 38.1 |
| NZ | 77.2 | 25.0 | 51.0 | 20.6 | 17.9 | 20.7 | 20.8 | 19.5 | 17.7 | 51.7 | 28.6 |
| UK | 56.8 | 20.8 | 32.7 | 18.9 | 17.1 | 19.0 | 18.9 | 17.1 | 18.6 | 35.0 | 20.9 |
| US | 64.0 | 32.9 | 45.0 | 31.7 | 29.1 | 30.6 | 28.5 | 29.2 | 30.6 | 49.3 | 33.4 |
| Total | 60.9 | 25.0 | 40.2 | 24.2 | 22.8 | 25.3 | 21.5 | 23.3 | 22.7 | 42.7 | 27.9 |

Numbers represent the % of respondents who attributed responsibility to individuals

**Table S5: Percentage of individuals attributing responsibility to the private sector stratified by country and policy**

| Country | Making sure healthy foods and beverages are available for everyone | Limiting the amount of junk-food advertising | Making healthy foods available in workplaces | Making sure healthy foods are affordable | Broadcasting public education campaigns about healthy eating | Deciding what nutrition information should be on food products | Deciding how many fast-food outlets are allowed in specific areas | Ensuring foods are reformulated to make them healthier | Setting targets for the amount of fat, sugar, and salt in packaged foods | Making sure children are not exposed to marketing for unhealthy foods | Making sure food production and distribution processes are environmentally friendly |
| --- | --- | --- | --- | --- | --- | --- | --- | --- | --- | --- | --- |
| Australia | 51.9 | 50.6 | 69.8 | 58.8 | 43.3 | 47.5 | 25.2 | 56.7 | 50.5 | 39.9 | 61.3 |
| Canada | 49.6 | 47.0 | 69.8 | 55.3 | 41.3 | 41.1 | 25.0 | 57.2 | 49.3 | 36.1 | 57.8 |
| China | 41.5 | 42.0 | 58.1 | 49.4 | 37.2 | 50.7 | 35.7 | 54.1 | 51.0 | 40.5 | 52.3 |
| India | 46.0 | 48.8 | 60.3 | 48.0 | 46.6 | 52.6 | 36.9 | 50.6 | 48.9 | 43.1 | 53.5 |
| NZ | 56.1 | 57.9 | 79.2 | 57.7 | 45.5 | 53.8 | 26.2 | 65.7 | 52.1 | 44.2 | 70.8 |
| UK | 50.7 | 46.8 | 67.7 | 56.9 | 38.6 | 49.3 | 25.3 | 56.3 | 43.6 | 41.8 | 57.0 |
| US | 44.7 | 44.9 | 63.3 | 54.8 | 46.9 | 43.6 | 27.2 | 51.1 | 47.8 | 36.6 | 54.3 |
| Total | 48.6 | 48.3 | 66.9 | 54.4 | 42.8 | 48.4 | 28.8 | 56.0 | 49.0 | 40.3 | 58.1 |

Numbers represent the % of respondents who attributed responsibility to the private sector

**Table S6: Percentage of individuals considering policies as not important stratified by country and policy**

| Country | Making sure healthy foods and beverages are available for everyone | Limiting the amount of junk-food advertising | Making healthy foods available in workplaces | Making sure healthy foods are affordable | Broadcasting public education campaigns about healthy eating | Deciding what nutrition information should be on food products | Deciding how many fast-food outlets are allowed in specific areas | Ensuring foods are reformulated to make them healthier | Setting targets for the amount of fat, sugar, and salt in packaged foods | Making sure children are not exposed to marketing for unhealthy foods | Making sure food production and distribution processes are environmentally friendly |
| --- | --- | --- | --- | --- | --- | --- | --- | --- | --- | --- | --- |
| Australia | 3.5 | 9.6 | 6.4 | 4.5 | 6.1 | 4.0 | 14.3 | 8.4 | 6.3 | 7.6 | 6.6 |
| Canada | 2.3 | 12.8 | 5.4 | 3.0 | 5.4 | 3.0 | 19.8 | 7.8 | 5.7 | 8.0 | 4.7 |
| China | 0.5 | 1.7 | 1.3 | 1.5 | 0.7 | 1.5 | 3.2 | 1.5 | 1.1 | 1.7 | 1.7 |
| India | 1.2 | 2.9 | 1.6 | 2.1 | 1.4 | 1.2 | 4.1 | 2.8 | 2.7 | 2.8 | 2.2 |
| NZ | 1.0 | 7.2 | 3.9 | 1.5 | 4.0 | 2.1 | 15.4 | 9.1 | 6.0 | 6.1 | 4.0 |
| UK | 2.8 | 7.2 | 5.1 | 3.2 | 4.2 | 2.9 | 9.8 | 8.2 | 5.5 | 6.1 | 5.1 |
| US | 3.3 | 14.7 | 5.8 | 4.8 | 6.9 | 3.9 | 26.8 | 10.9 | 8.6 | 12.0 | 6.7 |
| Total | 2.1 | 8.0 | 4.2 | 2.9 | 4.1 | 2.7 | 13.4 | 7.0 | 5.1 | 6.3 | 4.4 |

Numbers represent the % of respondents who considered policies as not important

**Table S7: Independent predictors for attributing responsibility for food policy to governments**

|  | Estimate | Standard Error | t value | 95% Confidence interval |
| --- | --- | --- | --- | --- |
| Gender (women vs men) | 0.146 | 0.079 | 1.850 | -0.009 to 0.300 |
| Age (per year) | -0.005 | 0.002 | -2.124 | -0.010 to 0.000^*^ |
| Income (middle vs lower third) | 0.001 | 0.098 | 0.015 | -0.191 to 0.194 |
| Income (upper vs lower third) | 0.226 | 0.115 | 1.959 | 0.000 to 0.451^*^ |
| University degree (yes vs no) | 0.186 | 0.022 | 8.436 | 0.143 to 0.229^*^ |
| Health (very good/good vs poor/fair) | -0.330 | 0.044 | -7.445 | -0.417 to -0.243^*^ |
| Healthy diet (yes vs no) | 0.280 | 0.112 | 2.494 | 0.060 to 0.501^*^ |

^*^ Statistically significant at p-value below 0.05
